# Cross-Species Translation of Biophase Half-Life and Potency of GalNAc-Conjugated siRNAs

**DOI:** 10.1089/nat.2022.0010

**Published:** 2022-12-05

**Authors:** Alessandro Boianelli, Yasunori Aoki, Maxim Ivanov, Anders Dahlén, Peter Gennemark

**Affiliations:** ^1^ Drug Metabolism and Pharmacokinetics, Research and Early Development, Cardiovascular, Renal and Metabolism (CVRM), BioPharmaceuticals R&D, AstraZeneca, Gothenburg, Sweden.; ^2^ Quantitative Biology SE, Data Sciences and Quantitative Biology, Discovery Sciences, AstraZeneca, Gothenburg, Sweden.; ^3^ Oligonucleotide Discovery, Discovery Sciences, BioPharmaceuticals R&D, AstraZeneca, Gothenburg, Sweden.; ^4^ Department of Biomedical Engineering, Linköping University, Linköping, Sweden.

**Keywords:** GalNAc-conjugated siRNA, KPD modeling, biophase half-life, translation

## Abstract

Small interfering RNAs (siRNAs) with *N*-acetylgalactosamine (GalNAc) conjugation for improved liver uptake represent an emerging class of drugs to treat liver diseases. Understanding how pharmacokinetics and pharmacodynamics translate is pivotal for *in vivo* study design and human dose prediction. However, the literature is sparse on translational data for this modality, and pharmacokinetics in the liver is seldom measured. To overcome these difficulties, we collected time-course biomarker data for 11 GalNAc–siRNAs in various species and applied the kinetic-pharmacodynamic modeling approach to estimate the biophase (liver) half-life and the potency. Our analysis indicates that the biophase half-life is 0.6–3 weeks in mouse, 1–8 weeks in monkey, and 1.5–14 weeks in human. For individual siRNAs, the biophase half-life is 1–8 times longer in human than in mouse, and generally 1–3 times longer in human than in monkey. The analysis indicates that the siRNAs are more potent in human than in mouse and monkey.

## Introduction

N-acetylgalactosamine (GalNAc)-conjugated small interfering RNAs (siRNAs) are double-stranded molecules containing a sense and an antisense strand, of which the latter elicits the pharmacological effect [1]. These drugs are designed to knock down a certain gene with complementary nucleotide sequences by degrading mRNA after transcription, and as an effect preventing translation. The GalNAc conjugation targets the siRNA specifically to the liver, and GalNAc–siRNAs represent an emerging class of drugs to treat various liver diseases [2].

Understanding how pharmacokinetics and pharmacodynamics translate between species is pivotal to set dose level and dosing schedule in preclinical proof-of-concept studies, to predict the human dose from preclinical data, and to set safety margins. Important translational data in this direction were recently reported in Ref. [3]. However, the literature is generally sparse on translational data for these GalNAc–siRNAs, and pharmacokinetics in the liver is seldom measured.

One way to learn more about translation of GalNAc–siRNAs is to estimate drug pharmacokinetics in the liver, target turnover, and siRNA potency from temporal biomarker data using mathematical dose–response modeling, so-called kinetic-pharmacodynamic (KPD) modeling [4]. Previous study in this direction on oligonucleotides mainly considered antisense oligonucleotides and human data [5]. In this study, we present an analysis of 11 GalNAc–siRNAs in several species. Specifically, we estimate biophase (liver) half-life and potency across species of GalNAc–siRNAs that have reached the clinical phase and for which data are available.

## Materials and Methods

We collected and digitized available literature time-course biomarker data from mice, monkeys (rhesus macaque *Macaca mulatta* or cynomolgus *Macaca fascicularis*), and humans of the following 11 GalNAc–siRNAs: Revusiran [6–8], Vutrisiran [9], Cemdisiran [10,11], Givlaari (Givosiran) [12,13], Lumasiran [14,15], Fitusiran [16,17], Inclisiran [18–20], ALN-HBV02 [21,22], ARO-APOC3 [23–26], ARO-ANG3 [27–30], and Olpasiran [31]. Compounds for which we could at least find relevant human data were included in the analysis. Group mean data were digitized using MATLAB (R2020a; The MathWorks, Natick, MA) or WebplotDigitizer (Ver. 4.5; Automeris, Pacifica, CA).

**FIG. 1.**
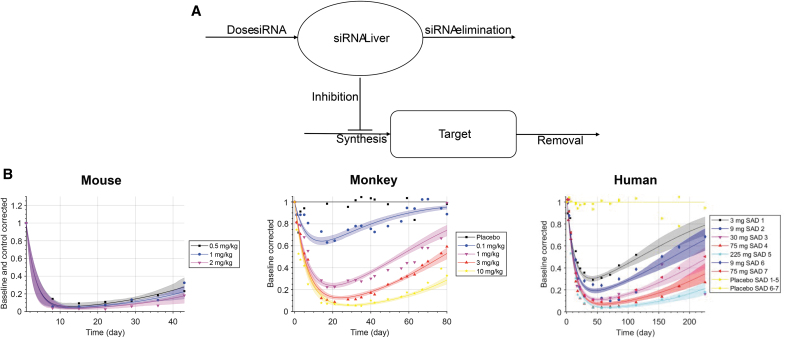
**(A)** Schematic diagram of the K-PD model. The amount of siRNA in the liver is governed by the dose and elimination rate. This amount inhibits the synthesis rate of the target according to the mode of action of siRNAs. Target is eliminated by its natural half-life. **(B)** Example of K-PD model fits to data for mouse, monkey, and human for different dose levels of Olpasiran. K-PD, kinetic-pharmacodynamic; siRNAs, small interfering RNAs.

We applied the K-PD modeling approach to estimate the biophase half-life in the target organ and the potency (*IDK*_50_, ie, the dose per unit of time that results in 50% reduction in the target mRNA or protein), see Fig. 1. The pharmacokinetics is described by a virtual one-compartment model aimed to represent the biophase. No drug concentration measurements are required, or available, and the model depends solely on the biomarker data for identification of all parameters. We used an indirect-response PD model, where the drug inhibits the zero-order synthesis rate *k_in_* of response.

In the model, the synthesis rate constant *k_S_* of response (*R*) is inhibited by a virtual infusion rate (*IR*), expressed in drug amount (*A*) per time unit, through a sigmoid Emax model:


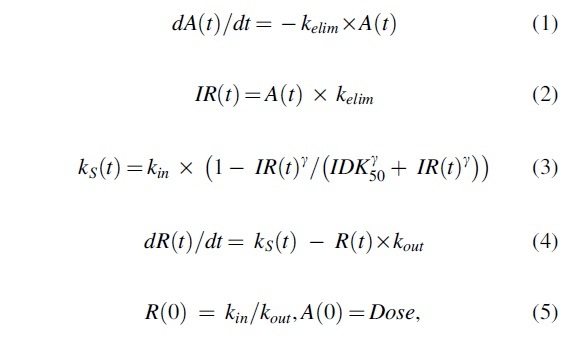


where *k_elim_* represents the elimination rate constant from the virtual compartment, *k_S_* and *k_out_* are the zero-order synthesis and the first-order degradation rate constants of the response *R*, *R*(0) is the baseline value of the response, *k_S_* is the time-dependent synthesis rate constant, *IDK*_50_ represents *IR* that leads to 50% inhibition of *k_S_*, *IDK*_50_ represents the apparent *in vivo* potency of the drug reflecting the ratio of clearance and bioavailability as well as the intrinsic potency of the drug, and γ is the Hill coefficient.

Numerical analyses were performed in MATLAB (R2020a; The MathWorks, Natick, MA). Specifically, the Matlab function *fminsearch* was used for solving the optimization problems encountered during parameter estimation. Parameter estimation was performed according to a maximum likelihood approach with an additive error model, using the naive-pooled data approach.

## Results and Discussion

Mouse, monkey, and human data were collected and digitized for 7, 8, and 11 of the considered GalNAc-conjugated siRNAs, respectively. Model parameters were generally well estimated in terms of confidence intervals (Table 1 and Supplementary Information). For example, Fig. 1B shows the data and model fit for Olpasiran. The analysis indicates that the biophase half-life of GalNAc-conjugated siRNAs is 0.6–3 weeks in mice, 1–8 weeks in monkey, and 1.5–14 weeks in humans.

**Table 1.** Parameter Estimation for N-Acetylgalactosamine Small Interfering RNAs

Species	K-PD model parameter	siRNA										
		Revusiran	Vutrisiran	Cemdisiran	Givlaari	Lumasiran	Fitusiran	Inclisiran	ALN-HBV02	ARO-APOC3	ARO-ANG3	Olpasiran
	Type			WT		AGXT KO	WT		WT	TG	WT	TG
Mouse	*IDK*_50__(mg/[kg·d])_			7.98e-3		2.41e-2	2.57e-2		6.79e-4	5.59e-3	4.86e-3	1.72e-5
	CI (5th, 95th Perc.)			(7.4e-3, 8.7e-3)		(1.5e-2, 4.9e-2)	(2.0e-2, 3.0e-2)		(4.5e-4, 9.2e-4)	(5.1e-3, 6.2e-3)	(3.1e-3, 5.6e-3)	(1.3e-6, 6.9e-4)
	PK *t*-half (d)			15.7		6.26	11.3		21.4	10.2	16.7	4.48
	CI (5th, 95th Perc.)			(14, 17)		(4.9, 12)	(7.5, 17)		(15, 34)	(8.3, 13)	(10, 39)	(3.2, 14)
	PD *t*-half (d)			2.11		12.6	1.19		1.54	1.82	1.17	1.64
	CI (5th, 95th Perc.)			(1.8, 3.4)		(5.8, 17)	(0.68, 2.3)		(1.2, 2.5)	(1.1, 2.2)	(0.4, 1.7)	(0.98, 2.3)
Monkey	*IDK*_50__(mg/[kg·d])_	0.281		7.06e-3	4.91e-2		1.36e-2	2.06e-2		3.09e-2	7.36e-3	4.79e-3
	CI (5th, 95th Perc.)	(0.20, 0.37)		(7.9e-6, 8.7e-3)	(4.9e-2, 5.1e-2)		(3.7e-3, 2.5e-2)	(1.5e-2, 2.5e-2)		(2.4e-2, 4.6e-2)	(7.5e-7, 1.6e-2)	(4.1e-3, 5.5e-3)
	PK *t*-half (d)	7.36		42.8	1.81		6.02	52.5		45.4	53.8	14.3
	CI (5th, 95th Perc.)	(5.6, 9.7)		(30, 4.9e4)	(1.7, 1.9)		(4.2, 7.4)	(34, 83)		(22, 80)	(12, 1.9e5)	(12, 17)
	PD *t*-half (d)	4.91		5.26	7.08		6.01	3.70		3.46	2.37	4.56
	CI (5th, 95th Perc.)	(3.9, 6.1)		(4.5, 6.3)	(6.9, 7.1)		(5.1, 7.1)	(2.4, 5.0)		(1.8, 4.8)	(0.5, 4.3)	(3.8, 5.2)
Human	*IDK*_50__(mg/[kg·d])_	0.217	3.06e-4	1.00e-3	5.57e-4	6.86e-3	3.20e-3	5.71e-3	2.52e-5	6.27e-4	4.00e-3	1.00e-4
	CI (5th, 95th Perc.)	(0.19, 0.25)	(2.5e-4, 3.6e-4)	(7.2e-4, 1.4e-3)	(3.1e-4, 6.8e-4)	(2.9e-3, 1.1e-2)	(2.2e-3, 4.5e-3)	(5.1e-3, 6.2e-3)	(6.1e-8, 1.6e-4)	(5.1e-4, 7.3e-4)	(3.5e-3, 4.5e-3)	(7.2e-5, 1.3e-4)
	PK *t*-half (d)	12.3	121	112	50.9	9.99	19.9	82.4	98.4	62.8	53.0	33.8
	CI (5th, 95th Perc.)	(10, 16)	(93, 163)	(99, 124)	(21, 130)	(6.6, 16)	(16, 25)	(73, 92)	(52, 8.1e4)	(50, 90)	(44, 66)	(27, 42)
	PD *t*-half (d)	4.82	8.16	4.98	5.25	15.9	12.3	4.07	9.49	3.79	4.43	8.41
	CI (5th, 95th Perc.)	(4.1, 5.4)	(7.0, 9.7)	(4.3, 5.7)	(0.60, 12)	(10, 21)	(9.5, 16)	(3.7, 4.5)	(7.2, 12)	(3.2, 4.5)	(3.7, 5.0)	(7.7, 9.2)

Summary of estimated parameters for the 10 GalNAc–siRNAs in mouse, monkey, and human. Body weight assumed 70 kg.

AGXT, alanine–glyoxylate aminotransferase-deficient; CI, confidence interval; d, day; KO, knockout; KPD, kinetic-pharmacodynamic; Perc., percentile; PK, pharmacokinetic; TG, transgenic; WT, wildtype.

**FIG. 2.**
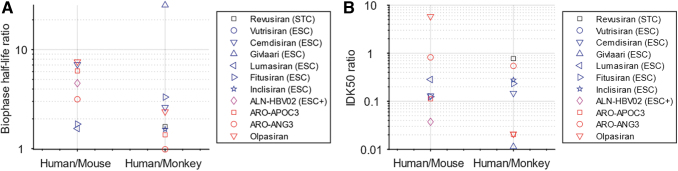
Predicted ratios between human and mouse and between human and monkey for biophase half-life **(A)** and potency *IDK*_50_ **(B)** for the analyzed GalNAc–siRNAs. *Black*: Alnylam's STC; *blue*: Alnylam's ESC; *magenta*: Alnylam's ESC-plus (ESC+); *red*: *arrowhead* Pharmaceuticals' chemistry. Biophase half-life was calculated as ln(2)/ *k_elim_*. ESC, enhanced stabilization chemistry; GalNAc, *N*-acetylgalactosamine; STC, standard template chemistry.

For individual siRNAs, the biophase half-life is 1–8 times longer in human than in mouse, and generally 1–3 times longer in human than in monkey (Fig. 2A). Givlaari deviates from the general pattern with a 28 times longer biophase half-life in human than in monkey. There is no clear dependency between translation of half-life between species and type of chemistry (indicated by the colors of the markers in Fig. 2A).

Potencies in form of *IDK*_50_ were relatively similar between mice and monkeys, and greater in humans. Specifically, *IDK*_50_ was predicted to be similar or smaller (up to 30-fold) in human than in mouse, and similar or smaller (up to 100-fold) in human than in monkey (Fig. 2B). Similarly to half-life, there is no clear dependency between translation of potency between species and type of chemistry. The higher potency observed in human is likely a result of optimization against the human sequence in the screening phase.

For two estimated half-lives, we could compare the results with previously reported data. First, the predicted biophase half-life of Fitusiran in human of ∼3 weeks compares well with the predicted liver half-life of 20 days in Ref. [32]. Second, the predicted biophase half-life of Givlaari in monkey of 1.8 days compares well with the predicted distributional half-life of 2.1 days from a two-compartment model fit to measured liver concentration data by Ref. [33].

The approach taken is limited by the lack of publicly available biomarker data in some species for certain siRNAs, and by the modeling on group mean data and not on individual data. The approach is supported by two comparisons with available reported data of biophase half-lives. The reported quantitative translational relationships may help guiding *in vivo* design and human dose predictions of GalNAc-conjugated siRNAs. In conclusion, we present the first systematic translational investigation of biophase (liver) half-life and potency of GalNAc–siRNAs.

## Supplementary Material

Supplemental data
