# Supplementary material for: Cross-Species Translation of Biophase Half-Life and Potency of GalNAc-Conjugated siRNAs
Source: Nucleic Acid Ther. 2022 Dec 5;32(6):507–12. doi: 10.1089/nat.2022.0010 (PMC9784597; doi:10.1089/nat.2022.0010)
Supplement: Supplemental data [file Supp_Data.pdf]

Supporting information

Cross-species translation of biophase half-life and potency of GalNAc-conjugated siRNAs

Alessandro Boianelli[1], Yasunori Aoki[1], Maxim Ivanov[3], Anders Dahlén[4], Peter Gennemark[1,2]

[1]Drug Metabolism and Pharmacokinetics, Research and Early Development, Cardiovascular, Renal and Metabolism (CVRM), BioPharmaceuticals R&D, AstraZeneca, Gothenburg, Sweden.

[2]Department of Biomedical Engineering, Linköping University, Linköping, Sweden

[3]Quantitative Biology SE, Data Sciences and Quantitative Biology, Discovery Sciences, AstraZeneca, Gothenburg, Sweden.

[4]Oligonucleotide Discovery, Discovery Sciences, BioPharmaceuticals R&D, AstraZeneca, Gothenburg, Sweden.

General structural information

The 3'-R group in Revusiran, Vutrisiran, Cemdisiran, Givosiran, Lumasiran, Fitusiran, Inclisiran and ALN-HBV02 corresponds to the GalNac-type shown below:

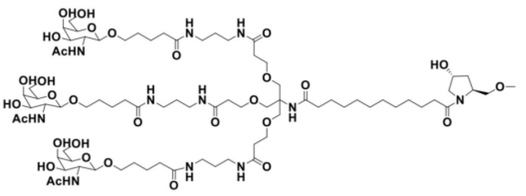

ARO-APOC3 and ARO-ANGPTL3 contain one inverted abasic building block on each end of the passenger strand. The 5'-R group in ARO-APOC3 and ARO-ANGPTL3 corresponds to the GalNac-type shown below:

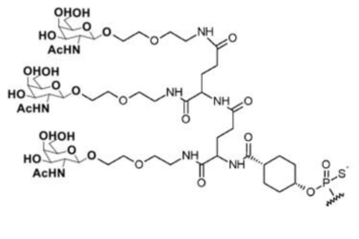

Olpasiran contain one inverted dA building block on 3’-end of passenger strand. The 5’-R group in Olpasiran corresponds to the GalNac-type shown below:

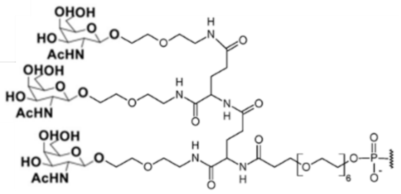

Revusiran

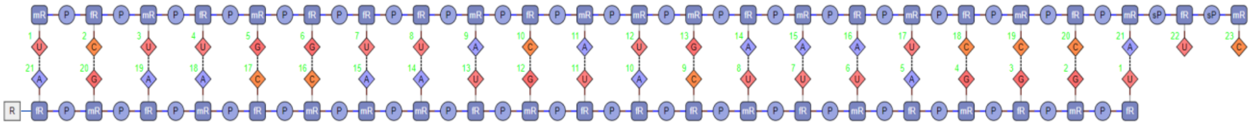

Biomarker: circulating TTR		
Chemistry: STC (Standard template chemistry)		
Sequence homology to cyno, rhesus and human		
Mouse	Monkey[1, 2]	Human[3]
	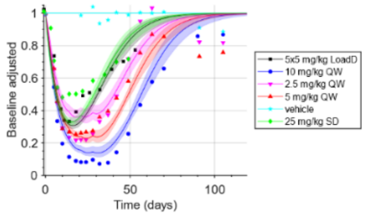	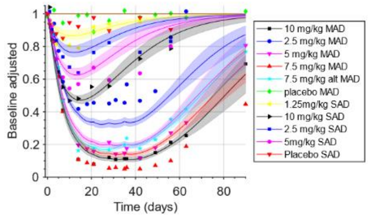
	Point prediction (5th and 95th percentiles)<br>kelim (1/days)=9.41e.2 (0.071, 0.13)<br>R0 (relative)=1 (fixed)<br>kout (1/days)=0.14 (0.11, 0.18)<br>**IDK50 (mg/kg/day)=0.281 (0.20 0.37)**<br>hill (adimensional)=1.02 (0.80, 1.3)<br>SIGMA_R (relative)=1.02e-2 (8.4e-3,1.2e-1)<br>**PK thalf (days)=7.36 (5.6,10)**<br>**PD thalf (days)=4.91 (3.9,6.1)**	Point prediction (5th and 95th percentiles)<br>kelim (1/day)=5.63e-02 (4.5e-02,6.8e-02)<br>R0 (relative)=1 (fixed)<br>kout (1/day)=0.143 (0.13, 0.17)<br>**IDK50 (mg/kg/day)=0.217 (0.19,0.25)**<br>hill (adimensional)=1.04 (0.9, 1.2)<br>SIGMA_R (relative)=8.30e-3 (5.9e-3,1.1e-2)<br>**PK thalf (days)=12.3 (10, 16)**<br>**PD thalf (days)=4.82 (4.1 5.4)**

Vutrisiran

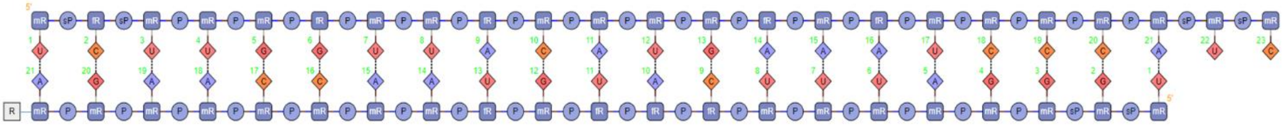

Biomarker: circulating TTR		
Chemistry: ESC (Enhanced stabilization chemistry)		
Sequence homology to cyno, rhesus and human		
Mouse	Monkey	Human[4]
		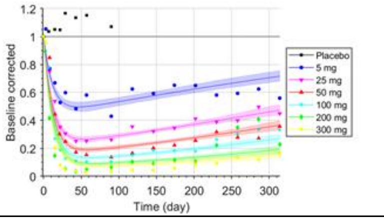
		Point prediction (5th and 95th percentiles)<br>kelim (1/day)=5.75e-03 (4.2e-03,7.3e-03)<br>R0 (relative)=1 (fixed)<br>kout (1/day)=8.50e-02 (7.1e-02, 9.9e-02)<br>**IDK50 (mg/kg/day)=3.06e-4 (2.5e-4, 3.6e-4)**<br>hill (adimensional)=0.641 (0.59, 0.70)<br>SIGMA_R (relative)=5.99e-03 (3.9e-3, 7.8e-3)<br>**PK thalf (day)=121 (93, 163)**<br>**PD thalf (day)=8.16 (7.0, 9.7)**

Cemdisiran

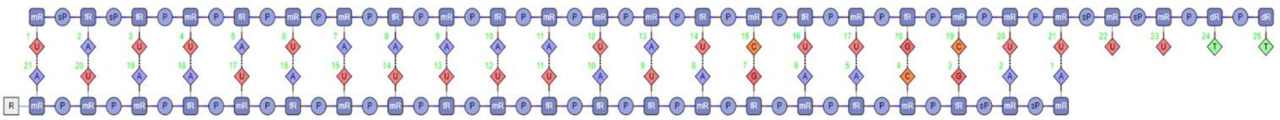

Biomarker: circulating C5 protein		
Chemistry: ESC (Enhanced stabilization chemistry)		
Sequence homology to cyno, rhesus, mouse and human		
Mouse[5]	Monkey[5]	Human[6]
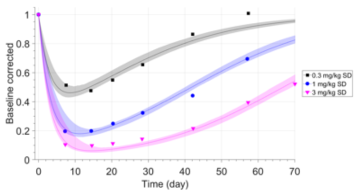	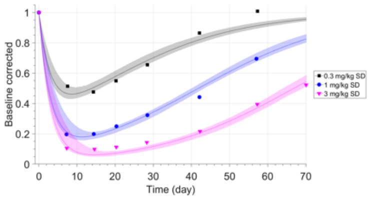	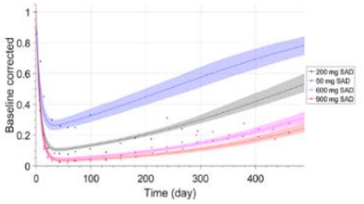
Point prediction (5th and 95th percentiles)<br>kelim (1/day)=4.39e-2(4.1e-2,4.8e-2)<br>R0 (relative)=1 (fixed)<br>kout (1/day)=0.328 (0.20, 0.37)<br>**IDK50 (mg/kg/day)=7.98e-3 (7.4e-3,8.7e-3)**<br>hill (adimensional)=1.25 (1.1, 1.4)<br>SIGMA_R (relative)=1.56e-3 (5.8e-4,2.2e-3)<br>**PK thalf (day)=15.7 (14, 17)**<br>**PD thalf (day)=2.11(1.8, 3.4)**	Point prediction (5th and 95th percentiles)<br>kelim (1/day)=1.61e-2 (1.3e-5, 2.3e-2)<br>R0 (relative)=1 (fixed)<br>kout (1/day)=0.131 (0.11, 0.16)<br>**IDK50 (mg/kg/day)=7.06e-3 (7.9e-6,8.7e-3)**<br>hill (adimensional)=0.907 (0.80, 1.1)<br>SIGMA_R (relative)=6.97e-3 (3.6e-3,9.2e-3)<br>**PK thalf (day)=42.8 (30, 4.9e4)**<br>**PD thalf (day)=5.26 (4.5, 6.3)**	Point prediction (5th and 95th percentiles)<br>kelim (1/day)=6.18e-3 (5.5e-3,7.0e-3)<br>R0 (relative)=1 (fixed)<br>kout (1/day)=0.139 (0.12, 0.16)<br>**IDK50 (mg/kg/day)=1.00e-3 (7.2e-4,1.4e-3)**<br>hill (adimensional)=0.830 (0.71, 0.96)<br>SIGMA_R (relative)=1.91e-3 (1.0e-3,2.5e-3)<br>**PK thalf (day)=111 (99, 124)**<br>**PD thalf (day)=4.98 (4.3,5.7)**

Givlaari (Givosiran)

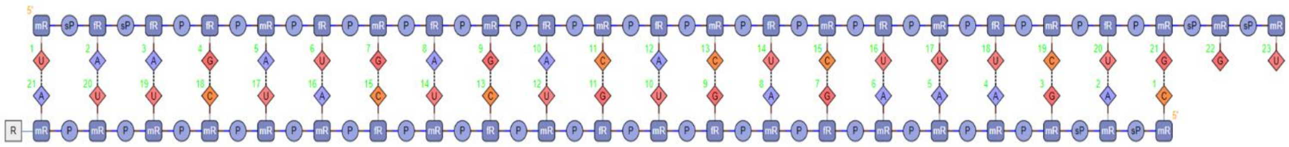

<table>
<tr><td colspan="3">Biomarker: circulating ALAS-1</td></tr>
<tr><td colspan="3">Chemistry: ESC (Enhanced stabilization chemistry)</td></tr>
<tr><td colspan="3">Sequence homology to cyno, rhesus, mouse and human</td></tr>
<tr><td>Mouse</td><td>Monkey[7]</td><td>Human[8]</td></tr>
<tr><td></td><td>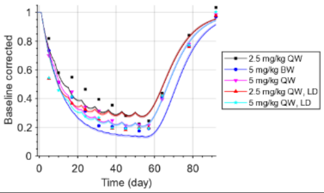</td><td>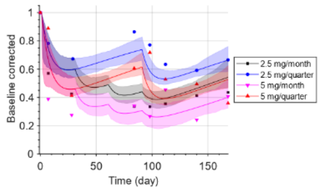</td></tr>
<tr><td></td><td><u>Point prediction (5th and 95th percentiles)</u>
kelim (1/day)=0.38 (0.3, 0.4)
R0 (relative)=1 (fixed)
kout (1/day)=9.77e-2 (9.0e-2, 1.0e-1)
IDK50 (mg/kg/day)=4.91e-2 (4.9e-2,5.1e-2)
hill (adimensional)=0.576 (0.57, 0.61)
SIGMA_R (relative)=1.50e-2 (6.3e-3, 1.6e-2)
PK thalf (day)=1.81 (1.7, 1.9)
PD thalf (day)=7.08 (6.9, 7.1)</td><td><u>Point prediction (5th and 95th percentiles)</u>
kelim (1/day)=1.36e-2 (5.2e-3,3.0e-2)
R0 (relative)=1 (fixed)
kout (1/day)=0.132 (5.6e-2, 1.2)
IDK50 (mg/kg/day)=5.57e-4 (3.1e-4, 6.8e-4)
hill (adimensional)=0.827 (0.51, 1.2)
SIGMA_R (relative)=1.51e-2 (7.0e-3,2.0e-2)
PK thalf (day)=50.9 (21, 130)
PD thalf (day)=5.25 (0.60, 12)</td></tr>
</table>

Lumasiran

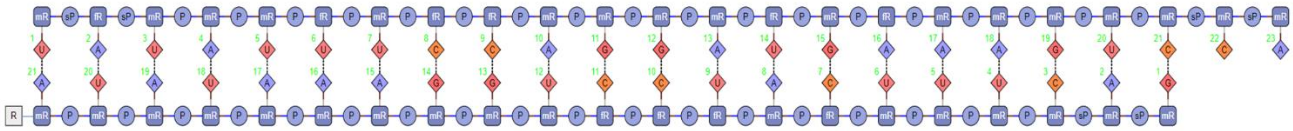

<table>
<tr><td colspan="3">Biomarker: circulating GO</td></tr>
<tr><td colspan="3">Chemistry: ESC (enhanced stabilization chemistry)</td></tr>
<tr><td colspan="3">Sequence homology to cyno, rhesus, mouse and human</td></tr>
<tr><td>AGXT KO mouse[9]</td><td>Monkey</td><td>Human[10]</td></tr>
<tr><td>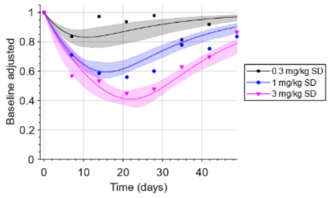</td><td></td><td>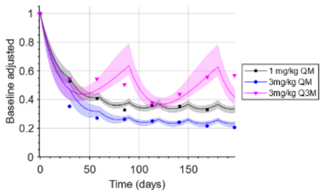</td></tr>
<tr><td><u>Point prediction (5th and 95th percentiles)</u>
Kelim (1/days)=1.10e-1 (5.7e-2,1.4e-1)
R0 (relative)=1 (fixed)
Kout (1/days)=5.47e-2 (4.1e-2,1.2e-1)
IDK50 (mg/kg/days)=2.41e-2 (1.5e-2,4.9e-2)
hill (adimensional)=1.91 (0.80, 5.3)
SIGMA_R (relative)=3.51e-3 (1.8e-3,5.4e-3)
PK thalf (days)=6.26 (4.9, 12)
PD thalf (days)=12.6 (5.8, 17)</td><td></td><td><u>Point prediction (5th and 95th percentiles)</u>
Kelim (1/days)=6.89e-2 (0.04.2e-2, 1.1e-1)
R0 (relative)=1 (fixed)
Kout (1/days)=4.32e-2 (3.3e-2,6.0e-2)
IDK50 (mg/kg/days)=6.86e-3 (2.9e-3,1.1e-2)
hill (adimensional)=0.478 (0.36, 0.59)
SIGMA_R (relative)=2.10e-3 (8.1e-4,4.0e-3)
PK thalf (1/days)=9.99 (6.6, 16)
PD thalf (1/days)=15.9 (10, 21)</td></tr>
</table>

Fitusiran

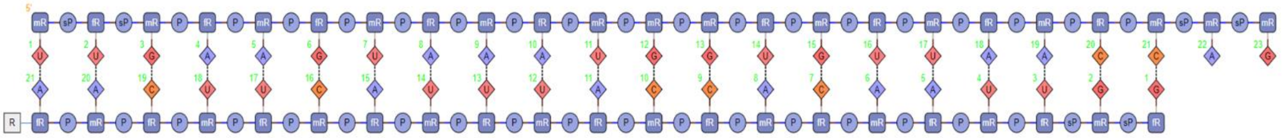

Biomarker: circulating antithrombin		
Chemistry: ESC (enhanced stabilization chemistry)		
Sequence homology to cyno, rhesus, mouse and human		
Mouse[11]	Monkey[11]	Human[12]
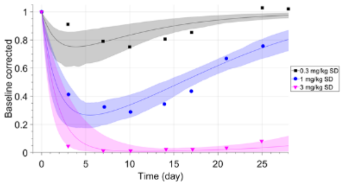	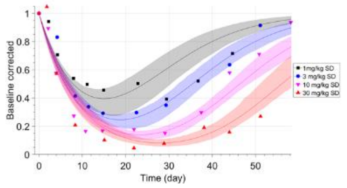	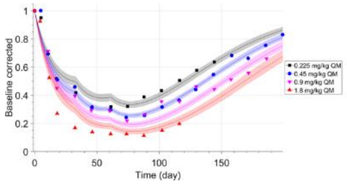
<u>Point prediction (5th and 95th percentiles)</u><br>kelim (1/day)=6.11e-2 (4.2e-2,9.2e-2)<br>R0 (relative)=1 (fixed)<br>kout (1/day)=0.581 (0.29, 0.98)<br>**IDK50 (mg/kg/day)=2.57e-2 (2.0e-2, 3.0e-2)**<br>hill (adimensional)= 1.92 (1.5, 2.7)<br>SIGMA_R (relative)=4.72e-3 (1.6e-3,5.4e-3)<br>**PK thalf (day)=11.3 (7.5, 17)**<br>**PD thalf (day)=1.19 (0.68, 2.3)**	<u>Point prediction (5th and 95th percentiles)</u><br>kelim (1/day)=0.11 (9.4e-2, 1.7e-1)<br>R0 (relative)=1 (fixed)<br>kout (1/day)=0.115 (0.098, 0.14)<br>**IDK50 (mg/kg/day)=1.36e-2 (3.7e-3,2.5e-2)**<br>hill (adimensional)= 1.04 (0.79, 1.5)<br>SIGMA_R (relative)=1.45e-2 (9.2e-3,1.8e-2)<br>**PK thalf (day)=6.02 (4.2, 7.4)**<br>**PD thalf (day)=6.01 (5.1, 7.1)**	<u>Point prediction (5th and 95th percentiles)</u><br>kelim (1/day)=3.46e-2 (2.7e-2,4.3e-2)<br>R0 (relative)=1 (fixed)<br>kout (1/day)=5.78e-2 (4.4e-2,7.3e-2)<br>**IDK50 (mg/kg/day)=3.20e-3 (2.2e-3,4.5e-3)**<br>hill(adim)= 0.690 (0.59, 0.86)<br>SIGMA_R (relative)=5.66e-3 (2.2e-3,9.3e-3)<br>**PK thalf (day)=19.9 (16, 25)**<br>**PD thalf (day)=12.3 (9.5, 16)**

Inclisiran

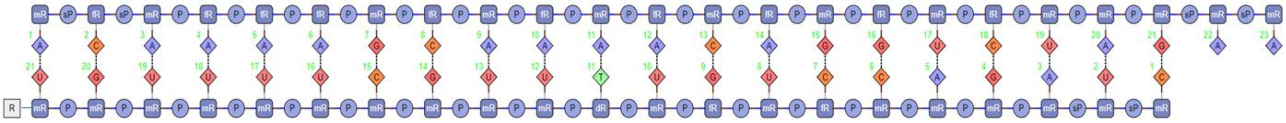

Biomarker: circulating PCSK9		
Chemistry: ESC (Enhanced stabilization chemistry)		
Sequence homology to rhesus and human[1]		
Mouse	Monkey[13, 14]	Human[15]
	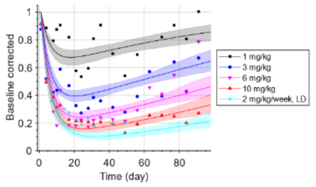	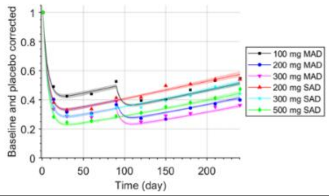
	<u>Point prediction (5th and 95th percentiles)</u><br>kelim (1/day)=1.32e-2 (8.0e-3, 2.0e-2)<br>R0 (relative)=1 (fixed)<br>kout (1/day)=0.187 (0.13, 0.28)<br>**IDK50 (mg/kg/day)=2.06e-2 (1.5e-2,2.5e-2)**<br>hill (adimensional)=1.08 (0.93, 1.3)<br>SIGMA_R (relative)=1.29e-2 (9.0e-3,2.0e-2)<br>**PK thalf (day)=52.5 (34, 83)**<br>**PD thalf (day)=3.70 (2.4, 5.0)**	<u>Point prediction (5th and 95th percentiles)</u><br>kelim (1/day)=8.41e-3 (7.5e-3,1.0e-2)<br>R0 (relative)=1 (fixed)<br>kout (1/day)=0.170 (0.15, 0.19)<br>**IDK50 (mg/kg/day)=5.71e-3 (5.1e-3, 6.2e-3)**<br>hill (adimensional)=0.574 (0.53, 0.62)<br>SIGMA_R (relative)=5.21e-4 (2.9e-4,7.0e-4)<br>**PK thalf (day)=82.4 (73, 92)**<br>**PD thalf (day)=4.07 (3.7, 4.5)**

[1]No hit in cyno due to incorrect annotation in genomic databases. Mismatch to mouse reported to be active in mouse because matches in the seed region see page 30 https://www.ema.europa.eu/en/documents/assessment-report/leqvio-epar-public-assessment-report_en.pdf "Sequence homology was sufficient to result in pharmacologic activity (LDL-C decreases) of inclisiran in all three species [rat, rabbit, mouse]." Reported to be active in cyno: "After single SC dosing inclisiran exhibited in female Cynomolgus monkeys a dose-dependent and sustainable reduction of PCSK9 protein paralleled by a lowering of serum LDL-C with similar kinetics."

ALN-HBV02

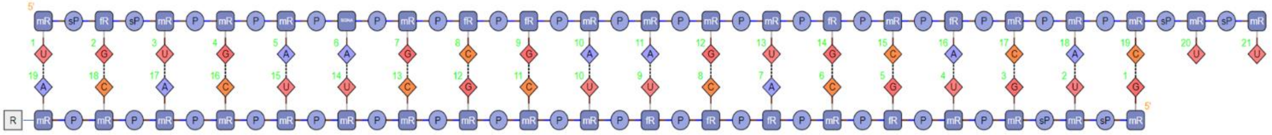

Biomarker: circulating Hepatitis B virus		
Chemistry: ESC+ (enhanced stabilization chemistry plus)		
Sequence homology (viral target)		
Mouse[16]	Monkey	Human[17]
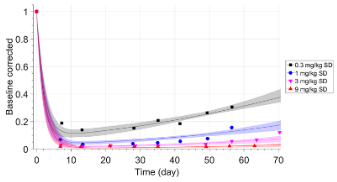		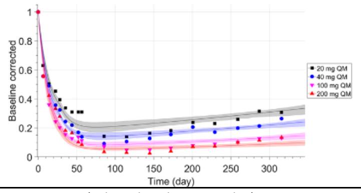
Point estimate (5th and 95th percentiles)<br>Kelim(1/day)=3.22e-2(2.0e-2,4.6e-2)<br>R0=1 (fixed)<br>Kout(1/day)=0.450 (0.28,0.57)<br>**IDK50(mg/kg/day)=6.79e-4 (4.5e-4,9.2e-4)**<br>hill (adimensional)=0.911 (0.81, 1.1)<br>SIGMA_R=3.61e-4 (1.2e-4,6.1e-4)<br>**PK thalf(day)=21.4 (15,34)**<br>**PD thalf(day)=1.53 (1.2,2.5)**		Point estimate (5th and 95th percentiles)<br>Kelim(1/day)=6.92e-3 (8.3e-6,1.4e-2)<br>R0=1 (fixed)<br>kout=7.29e-2 (5.8e-2,9.5e-2)<br>**IDK50(mg/kg/day)=2.52e-5 (6.2e-8,1.6e-4)**<br>hill (adimensional)=0.416 (0.26, 0.61)<br>SIGMA_R=1.78e-3 (1.1e-4,2.7e-3)<br>**PK thalf(day)=98.4 (52,8.1e4)**<br>**PD thalf(day)=9.49 (7.2, 12)**

ARO-APOC3

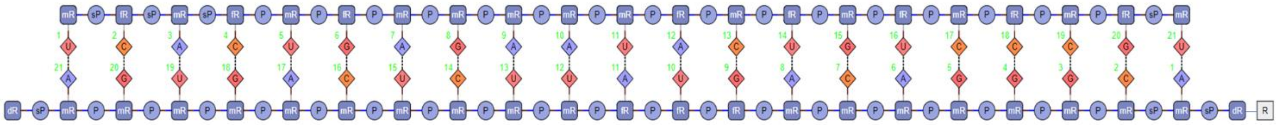

Biomarker: circulating APOC-3		
Chemistry: Arrowhead		
Sequence homology to cyno, rhesus, TG mouse and human		
TG Mouse[18]	Monkey[18, 19]	Human[20, 21]
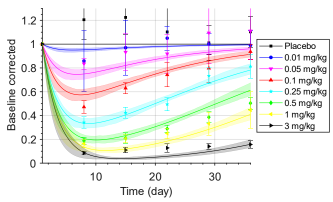	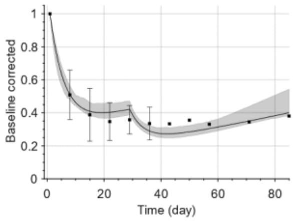	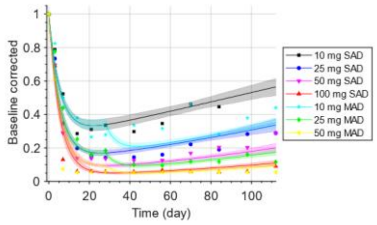
Point prediction (5th and 95th percentiles)<br>kelim (1/day)=6.74e-2 (5.5e-2,8.4e-2)<br>R0 (relative)=1 (fixed)<br>kout (1/day)=0.379 (0.30, 0.58)<br>**IDK50 (mg/kg/day)=5.59e-3 (5.1e-3,6.2e-3)**<br>hill (adimensional)=1.19 (1.1, 1.4)<br>SIGMA_R (relative)=5.67e-3 (3.5e-2,7.4e-3)<br>**PK thalf (day)=10.2 (8.3, 13)**<br>**PD thalf (day)=1.82 (1.1, 2.2)**	Point prediction (5th and 95th percentiles)<br>kelim (1/day)=1.52e-2 (8.2e-3,3.1e-2)<br>R0 (relative)=1 (fixed)<br>kout (1/day)=0.200 (0.14, 0.37)<br>**IDK50 (mg/kg/day)=3.09e-2 (2.4e-2,4.6-2)**<br>hill (adimensional)=1.00 (1.0, 1.0)<br>SIGMA_R (relative)=1.76e-3 (3.2e-4,2.5e-3)<br>**PK thalf (day)=45.4 (22, 80)**<br>**PD thalf (day)=3.46 (1.8, 4.8)**	Point prediction (5th and 95th percentiles)<br>kelim (1/day)=1.10e-2 (7.5e-3,1.4e-2)<br>R0 (relative)=1 (fixed)<br>kout (1/day)=0.182 (0.15, 0.21)<br>**IDK50 (mg/kg/day)=6.27e-4(5.1e-4, 7.3e-4)**<br>hill (adimensional)=1.02 (0.95, 1.2)<br>SIGMA_R (relative)=4.15e-3 (2.4e-3,5.4e-3)<br>**PK thalf (day)=62.8 (50, 90)**<br>**PD thalf (day)=3.79 (3.2, 4.5)**

ARO-AGPT3

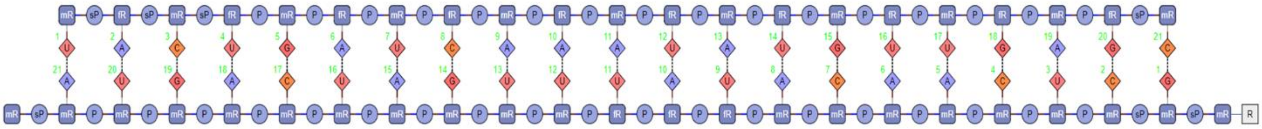

Biomarker: circulating ANGPTL-3[2]		
Chemistry: Arrowhead		
Sequence homology to mouse, rhesus, cyno and human		
Mouse[22]	Monkey[22]	Human[23-25]
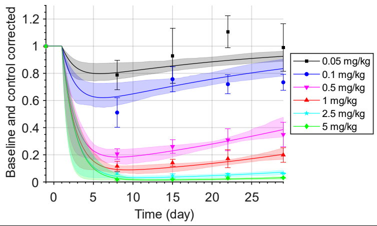	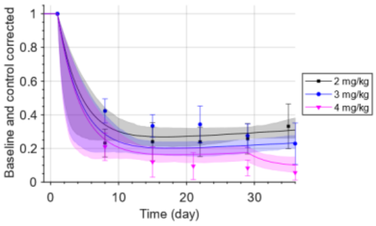	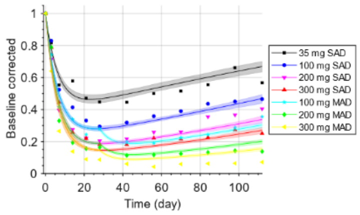
Point prediction (5th and 95th percentiles)<br>kelim (1/day)=4.13e-2 (1.7e-2,6.5e-2)<br>R0 (relative)=1 (fixed)<br>kout (1/day)=0.592 (0.41, 1.6)<br>**IDK50 (mg/kg/day)=4.86e-3(3.1e-3,5.7e-3)**<br>hill (adimensional)=1.29 (1.1, 1.6)<br>SIGMA_R (relative)=3.01e-3 (7.2e-4, 4.7e-3)<br>**PK thalf (day)=16.7 (10, 39)**<br>**PD thalf (day)=1.17 (0.43, 1.7)**	Point prediction (5th and 95th percentiles)<br>kelim (1/day)=0.012879 (7.0e-7, 4.7e-2)<br>R0 (relative)=1 (1, 1)<br>kout (1/day)=0.292 (0.15, 1.3)<br>**IDK50 (mg/kg/day)=7.36e-3 (7.5e-07, 1.6e-2)**<br>hill (adimensional)=0.952 (0.53, 1.7)<br>SIGMA_R (relative)=4.42e-3 (1.5e-3,5.9e-3)<br>**PK thalf (day)=53.8 (12, 1.9e5)**<br>**PD thalf (day)=2.37(0.56, 4.3)**	Point prediction (5th and 95th percentiles)<br>kelim (1/day)=1.30e-2 (1.0e-2,1.6e-2)<br>R0 (relative)=1 (fixed)<br>kout (1/day)=0.15 (0.13, 0.18)<br>**IDK50 (mg/kg/day)=4.00e-3 (3.5e-3, 4.5e-3)**<br>hill (adimensional)=0.789 (0.73, 0.86)<br>SIGMA_R (relative)=2.51e-3 (1.8e-3,3.1e-3)<br>**PK thalf (day)=53.0 (44, 66)**<br>**PD thalf (day)=4.43 (3.7, 5.0)**

[2]CACNA2D1 and ANGPTL-3 both predicted to be likely targeted. CACNA2D1 has sequence mismatches but some of these constitute wobble pairs that gives a favorable delta G and are valid in RNA to RNA interactions. Therefore character based alignment based methods gives different results from RISearch2 which is tailored to specifically consider RNA:RNA interactions and thermodynamics of hybridization. ANGPTL-3 is mismatched in the first and last base of the guide sequence. Experimental off target assessment can assess if this in silico off target is knocked down in vivo

Olpasiran

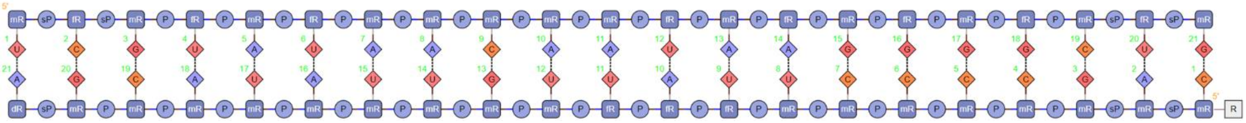

<table>
<tr><td colspan="3">Biomarker: circulating Lp(a)</td></tr>
<tr><td colspan="3">Chemistry: Arrowhead</td></tr>
<tr><td colspan="3">Sequence homology to rhesus, cyno and human</td></tr>
<tr><td>Mouse[26]</td><td>Monkey[26]</td><td>Human[26]</td></tr>
<tr><td>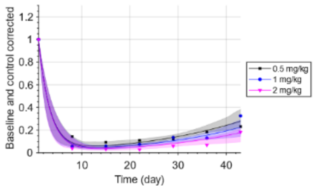</td><td>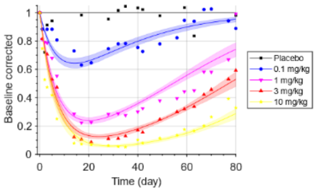</td><td>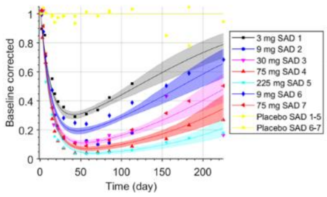</td></tr>
<tr><td><u>Point prediction (5th and 95th percentiles)</u>
kelim (1/day)=1.5e-1 (4.0e-2,3.0e-1)
R0 (relative)=1 (1, 1)
kout (1/day)=0.422 (0.30, 0.70)
IDK50 (mg/kg/day)=1.72e-5 (1.3e-6, 7.0e-4)
hill (adimensional)=0.419 (0.31, 0.78)
SIGMA_R (relative)=0.00117 (1.0e-4,1.7e-3)
PK thalf (day)=4.48 (3.2, 14)
PD thalf (day)=1.64 (0.98, 2.3)</td><td><u>Point prediction (5th and 95th percentiles)</u>
kelim (1/day)=4.83e-2 (4.0e-2,6.0e-2)
R0 (relative)=1 (1, 1)
kout (1/day)=0.152 (0.13, 0.19)
IDK50 (mg/kg/day)=4.79e-3 (4.1e-3,6.0e-3)
hill (adimensional)=0.864 (0.79, 1.0)
SIGMA_R (relative)=7.82e-3 (5.2e-3,1.2e-3)
PK thalf (day)=14.3 (12, 17)
PD thalf (day)=4.56 (3.8, 5.2)</td><td><u>Point prediction (5th and 95th percentiles)</u>
kelim (1/day)=2.05e-2 (1.0e-2, 3.0e-2)
R0 (relative)=1 (1, 1)
kout (1/day)=8.23e-2 (7.0e-2, 1.0e-1)
IDK50 (mg/day)= 1.00e-4 (7.2e-5,1.3e-4)
hill (adimensional)=0.610 (0.53, 0.70)
SIGMA_R (relative)=5.92e-3 (4.1e-3, 8.0e-3)
PK thalf (day)=33.8 (27, 42)
PD thalf (day)=8.41 (7.7, 9.2)</td></tr>
</table>

References

1. Butler JS, A Chan, S Costelha, S Fishman, JL Willoughby, TD Borland, S Milstein, DJ Foster, P Goncalves, et al. (2016). Preclinical evaluation of rnai as a treatment for transthyretin-mediated amyloidosis. Amyloid **23**: 109-118.
2. Rajeev K, T Zimmermann, M Manoharan, M Maier, S Kuchimanchi, and K Charisse. (2014). Rnai agents, compositions and methods of use thereof for treating transthyretin (ttr) associated diseases. WO2015042564A1
3. Zimmermann TS, V Karsten, A Chan, J Chiesa, M Boyce, BR Bettencourt, R Hutabarat, S Nochur, A Vaishnaw, et al. (2017). Clinical proof of concept for a novel hepatocyte-targeting galnac-sirna conjugate. Mol Ther **25**: 71-78.
4. Habtemariam BA, V Karsten, H Attarwala, V Goel, M Melch, VA Clausen, P Garg, AK Vaishnaw, MT Sweetser, et al. (2021). Single-dose pharmacokinetics and pharmacodynamics of transthyretin targeting n-acetylgalactosamine-small interfering ribonucleic acid conjugate, vutrisiran, in healthy subjects. Clin Pharmacol Ther **109**: 372-382.

5. Kusner LL, K Yucius, M Sengupta, AG Sprague, D Desai, T Nguyen, K Charisse, S Kuchimanchi, R Kallanthottathil, et al. (2019). Investigational rnai therapeutic targeting c5 is efficacious in pre-clinical models of myasthenia gravis. Mol Ther Methods Clin Dev **13**: 484-492.
6. Badri P, X Jiang, A Borodovsky, N Najafian, J Kim, VA Clausen, V Goel, B Habtemariam, and G Robbie. (2021). Pharmacokinetic and pharmacodynamic properties of cemdisiran, an rnai therapeutic targeting complement component 5, in healthy subjects and patients with paroxysmal nocturnal hemoglobinuria. Clinical Pharmacokinet **60**: 365-378.
7. EMA. (2020). Assessment report. Givlaari international non-proprietary name: Givosiran. EMEA/H/C/004775/0000
8. Sardh E, P Harper, M Balwani, P Stein, D Rees, DM Bissell, R Desnick, C Parker, J Phillips, et al. (2019). Phase 1 trial of an rna interference therapy for acute intermittent porphyria. N Engl J Med **380**: 549-558.
9. EMA. (2020). Assessment report oxlumo. EMA/568312/2020
10. Erbe DV. (2020). Methods for inhibition of hao1 (hydroxyacid oxidase 1 (glycolate oxidase)) gene expression. WO2019014491A1
11. Sehgal A, S Barros, L Ivanciu, B Cooley, J Qin, T Racie, J Hettinger, M Carioto, Y Jiang, et al. (2015). An rnai therapeutic targeting antithrombin to rebalance the coagulation system and promote hemostasis in hemophilia. Nat Med **21**: 492-497.
12. Pasi KJ, S Rangarajan, P Georgiev, T Mant, MD Creagh, T Lissitchkov, D Bevan, S Austin, CR Hay, et al. (2017). Targeting of antithrombin in hemophilia a or b with rnai therapy. N Engl J Med **377**: 819-828.
13. Borodovsky A, W Querbes, J Sutherland, R Hutabarat, S Milstein, S Kuchimanchi, R Kuchimanchi, K Charisse, K Yucius, et al. (2014). Development of monthly to quarterly subcutaneous administration of rnai therapeutics targeting the metabolic diseases genes pcsk9, apoc3 and angptl3. Circulation **130**: A11936-A11936.
14. Borodovsky A, RG Kallanthottathil, K Fitzgerald, M Frank-Kamenetsky, W Querbes, M Maier, K Charisse, S Kuchimanchi, M Manoharan, et al. (2014). Pcsk9 sirna compositions and methods of use thereof. WO2014089313A1
15. Ray KK, U Landmesser, LA Leiter, D Kallend, R Dufour, M Karakas, T Hall, R Troquay, T Turner, et al. (2017). Inclisiran in patients at high cardiovascular risk with elevated ldl cholesterol. N Engl J Med **376**: 1430-1440.
16. Stuart M, N Tuyen, A Castoreno, A Liebow, J Vasant, M Maier, and L Sepp-Lorenzino. Preclinical development of an rnai therapeutic drug candidate targeting hepatitis b virus. 2017 https://www.alnylam.de/wp-content/uploads/2017/09/OTS-2017_Poster-4_Milstein-et-al..pdf. Accessed January 27, 2022
17. Gane E, Y-S Lim, D Cloutier, L Shen, A Cathcart, X Ding, P Pang, S Huang, and R Yuen. Safety and antiviral activity of vir-2218, an x-targeting rnai therapeutic, in participants with chronic hepatitis b infection: Week 48 follow-up results. https://investors.vir.bio/static-files/10c2cdad-2e8a-49c1-b8f7-a11d8906bc26. Accessed March 4, 2022
18. Li Z, Z Rui, T Pei, S Kanner, and S Wong. (2019). Rna interference agents and compositions for inhibiting the expression of apolipoprotein c-iii (apoc3). WO2019051402A1
19. Wong SC, Z Li, B Given, M Seefeld, A Andersen, R Zhu, P Havel, J Hamilton, J Graham, et al. (2019). Personalized medicine for dyslipidemias by rna interference-mediated reductions in apolipoprotein c3 or angiopoietin-like protein 3. J Clin Lipid **13**: e15.
20. Schwabe C, R Scott, DR Sullivan, J Baker, P Clifton, J Hamilton, B Given, S Melquist, J Knowles, et al. (2019). Rna interference targeting apolipoprotein c-iii results in deep and prolonged reductions in plasma triglycerides. Circulation **140**: E987-E987.
21. Clifton P, D Sullivan, J Baker, C Schwabe, S Thackwray, R Scott, J Hamilton, T Chang, B Given, et al. (2020). Pharmacodynamic effect of aro-apoc3, an investigational hepatocyte-targeted rna interference therapeutic targeting apolipoprotein c3, in patients with hypertriglyceridemia and multifactorial chylomicronemia. Circulation **142**: A12594-A12594.

22. Li Z, R Zhu, and S Wong. (2019). Rnai agents and compositions for inhibiting expression of angiopoietin-like 3 (angptl3), and methods of use. WO2019055633A1
23. Watts GF, C Schwabe, R Scott, P Gladding, D Sullivan, J Baker, P Clifton, J Hamilton, B Given, et al. (2020). Pharmacodynamic effect of aro-ang3, an investigational rna interference targeting hepatic angiopoietin-like protein 3, in patients with hypercholesterolemia. Circulation **142**: A15751-A15751.
24. Watts G, C Schwabe, R Scott, P Gladding, D Sullivan, J Baker, P Clifton, J Hamilton, B Given, et al. (2020). Rnai inhibition of angiopoietin-like protein 3 (angptl3) with aro-ang3 mimics the lipid and lipoprotein profile of familial combined hypolipidemia. Eur Heart J **41**: ehaa946. 3331.
25. Watts GF, C Schwabe, R Scott, P Gladding, DR Sullivan, J Baker, P Clifton, J Hamilton, B Given, et al. (2019). Rna interference targeting hepatic angiopoietin-like protein 3 results in prolonged reductions in plasma triglycerides and ldl-c in human subjects. Circulation **140**: E987-E988.
26. Koren MJ, PM Moriarty, SJ Baum, J Neutel, M Hernandez-Illas, HS Weintraub, M Florio, H Kassahun, S Melquist, et al. (2022). Preclinical development and phase 1 trial of a novel sirna targeting lipoprotein(a). Nat Med **28**: 96-103.
